# Transposon Mutagenesis in *Bifidobacterium breve*: Construction and Characterization of a Tn5 Transposon Mutant Library for *Bifidobacterium breve* UCC2003

**DOI:** 10.1371/journal.pone.0064699

**Published:** 2013-05-30

**Authors:** Lorena Ruiz, Mary O’Connell Motherway, Noreen Lanigan, Douwe van Sinderen

**Affiliations:** Department of Microbiology and Alimentary Pharmabiotic Centre, National University of Ireland, Cork, Ireland; University of Florida, United States of America

## Abstract

Bifidobacteria are claimed to contribute positively to human health through a range of beneficial or probiotic activities, including amelioration of gastrointestinal and metabolic disorders, and therefore this particular group of gastrointestinal commensals has enjoyed increasing industrial and scientific attention in recent years. However, the molecular mechanisms underlying these probiotic mechanisms are still largely unknown, mainly due to the fact that molecular tools for bifidobacteria are rather poorly developed, with many strains lacking genetic accessibility. In this work, we describe the generation of transposon insertion mutants in two bifidobacterial strains, *B. breve* UCC2003 and *B. breve* NCFB2258. We also report the creation of the first transposon mutant library in a bifidobacterial strain, employing *B. breve* UCC2003 and a Tn5-based transposome strategy. The library was found to be composed of clones containing single transposon insertions which appear to be randomly distributed along the genome. The usefulness of the library to perform phenotypic screenings was confirmed through identification and analysis of mutants defective in D-galactose, D-lactose or pullulan utilization abilities.

## Introduction

Members of the genus *Bifidobacterium* are anaerobic Gram-positive bacteria, constituting one of the members of Actinobacteria phylum that inhabit the human gastrointestinal tract (GIT) [1], [2]. Bifidobacteria represent one of the dominant bacterial commensals in the infant gut, where they can be present at concentrations of 10^10^ cells per gram of feces, while they are less abundant in adults and elderly people [3], [4], [5].

The probiotic concept was first introduced by Metchnikoff [6], while probiotics are currently defined by the WHO as “live microorganisms which when administered in adequate amounts confer a health benefit on the host” [7]. A range of studies support the idea that bifidobacteria play a significant role in maintaining the health status of the host, by eliciting a plethora of beneficial properties, such as immune modulation, protection against infectious diseases, reduction of cholesterol levels, alleviation of lactose intolerance, reducing the symptoms of inflammatory disorders of the GIT, and production of vitamins and acetate [8], [9], [10], [11]. Therefore, some bifidobacterial strains are commercially exploited as probiotics (i.e. *B. animalis* subsp. *lactis* Bb12, *B. animalis* subsp. *lactis* Bi-07 300B, *Bifidobacterium animalis* subsp. *lactis* DN-173 010, or *B. longum* Bb536), being widely included in functional foods [10], [12]. Moreover, their status as “generally recognized as safe” (GRAS) organisms makes them potential candidates for the development of delivery systems to produce peptides or proteins of interest at intestinal level as well as for the use as live oral vaccines [13], [14], [15], [16].

During the last decade, 37 complete bifidobacterial genome sequences have become publicly available, with an additional 14 incomplete genomes and targeted sequencing projects currently listed on the Integrated Microbial Genomes Database (http://img.jgi.doe.gov). These genome-scale analyses have contributed significantly to our understanding as to how these bacteria colonize and adapt to the human gut [17], [18], [19], [20]. Nevertheless, until recently bifidobacteria have been rather recalcitrant to genetic manipulation, which represents one of the main reasons for our poor understanding of the molecular mechanisms underlying their probiotic attributes. Development of molecular methodologies that facilitate genetic accessibility is thus critical to allow functional genomic analyses of bifidobacteria.

In molecular biology, the gold standard method to determine the function of predicted genes and establish which genes are essential under particular environmental conditions is to disrupt the gene and determine the phenotypic change. Transformation efficiencies of individual bifidobacterial strains are generally too low to generate targeted mutants by homologous recombination. A well-characterized exception to this is *Bifidobacterium breve* UCC2003, where this limitation was overcome by methylation of a non-replicating plasmid to by-pass the restriction-modification systems and to force direct and homology-directed chromosomal integration into a targeted gene [21]. In addition, a conditionally replicating plasmid was recently described for a bifidobacterial strain [22]. Although a double cross-over recombination methodology has been reported for a *Bifidobacterium longum* strain, this procedure is tedious and highly strain dependent [23]. Remarkably, only targeted mutagenesis systems have been reported in bifidobacteria and we are not aware of any random-mutagenesis technology applicable to bifidobacteria.

Transposons are a powerful tool in molecular biology research and have been widely used to create mutant libraries in a wide range of genera [24], [25], [26], [27]. Such a mutant library is highly valuable as it allows high through-put screening aimed at the identification of genes essential for defined phenotypes [28]. In the present study we describe the implementation of a Tn5-based transposon mutagenesis system in two different bifidobacterial strains, *B. breve* UCC2003 and *B. breve* NCFB2258, for which relatively high transformation efficiencies have previously been achieved [21], [29]. We also report the creation of a collection of nearly 20,000 transposon insertion mutants in our model strain *B. breve* UCC2003 which, to our knowledge, represents the first genome-wide random mutagenesis approach for bifidobacteria. Analysis of transposon insertion mutants by Southern hybridization and sequencing of transposon insertion sites confirmed non-biased transposon insertion events. Phenotypic screenings for growth deficiencies in certain carbohydrates further allowed the validation of the usefulness of this mutant bank and revealed in most cases a direct and logical correlation between a particular growth-deficient phenotype and the mutation of a specific gene.

## Materials and Methods

### Bacterial Strains and Growth Conditions

*B. breve* UCC2003 and *B. breve* NCFB2258 were grown at 37°C in RCM (Oxoid, Hamphsire, England) or modified de Man Rogosa and Sharpe (mMRS) medium, made from first principles [30], supplemented with 0.05% L-cysteine (Sigma, St. Louis, USA) and 1% w/v of a specific carbohydrate as the sole carbon and energy source in an anaerobic chamber (Mac 500, Don Whitley Scientific, West Yorkshire, United Kingdom). *E. coli* EC101 was grown at 37°C in Luria Bertani (LB) broth. Where appropriate, 10 µg ml-1 of tetracycline (Tet) or 50 µg ml-1 of kanamycin (Kan) was added for transposon or plasmid selection. D-Glucose, D-Galactose, D-Lactose and D-Ribose were purchased from Sigma, Pullulan was obtained from Hayashibara (Japan) and Vivinal GOS was from FrieslandCampina (Amersfoort, The Netherlands).

### Construction of a Tetracycline Resistant Tn5 Transposon

Extensor Master Mix Polymerase (Thermo Fisher Scientific, Waltham, Massachusetts) was used for all PCR reactions. A tetracycline resistance cassette of bifidobacterial origin, *tetW* [31], was PCR amplified from pAM5 using the primer pair TetWFw and TetWRev (Table 1). The amplified product was sequentially restricted with SphI and XbaI, and ligated into similarly digested pMOD2 (Epicentre Biotechnologies, Madison, USA), yielding pMOD2-TetW. Preparation of *E. coli* electrocompetent cells and electrotransformation were performed according to previously described procedures [32]. *E. coli* transformants were selected on LB agar plates supplemented with 50 µg ml-1 of kanamycin and 10 µg ml-1 of tetracycline. Presence of the correct construct among the transformants was verified through restriction mapping and subsequent sequencing (Eurofins MWG Operon, Ebersberg, Germany).

**Table 1:** Strains, plasmids and primers used.

Strains	Relevant phenotype or genotype	Reference or Source
*B. breve* UCC2003	Infant isolate	[37]
*B. breve* NCFB2258	Infant isolate	NCFB
*E. coli* EC101	Cloning host, repA^+^, Kan^r^	[59]
**Plasmids**		
pMOD2	Source of tn5 terminal ends, Amp^R^	Epicentre Biotechnology
pMOD2-TetW	Tet^R^ derivative of pMOD2	This study
**Primers**	**Sequence**	
TetW-Fw	5′-CGCTAGTCTAGAGCTCATGTACGGTAAGGAAG-3′	This study
TetW-Rev	5′CGCTAGGCATGC **AAAACCCTCGGTCGGTCTGACCGGGGGTTTT**GATTACATTACCTTCTGAAACATATGGC-3′	This study
pMOD<MCS>Fw	5′-ATTCAGGCTGCGCAACTGT-3′	Epicentre Biotechnology
pMOD<MCS>Rev	5′-GTCAGTGAGCGAGGAAGCGGAAG-3′	Epicentre Biotechnology
i-PCR-Fw	5′-GCATACCGTACTGATCTG-3′	This study
i-PCR-Rev	5′-CAATCATACCGGCTTCC-3′	This study
pMOD-fw-seq	5′-GCCAACGACTACGCACTAGCC-3′	Epicentre Biotechnology
pMOD-rev-seq	5′-GAGCCAATATGCGAGAACACC-3′	Epicentre Biotechnology

Restriction sites are underlined and a rho-transcriptional terminator sequence is highlighted in bold. NCFB, National Collection of Food Bacteria.

Primers pMOD<MCS>Fw and pMOD<MCS>Rev (Table 1) were used to PCR amplify a TetW-Tn5-encompassing transposon (EZ::TN<TetW>) from pMOD2-TetW. The transposon encompasses *tetW* and a rho-independent transcriptional terminator, flanked by the 19 bp terminal ends recognized by the Tn5 transposase [33]. The transcriptional terminator is identical in sequence to the region located immediately downstream of *clp*P in *B. breve* UCC2003 [34]. Transposon ends were pruned by PshAI digestion according to manufactureŕs instructions (Epicentre Biotechnologies, Madison, USA). Pruned transposon was subsequently purified using high pure PCR purification columns (Roche, West Sussex, United Kingdom), ethanol precipitated and concentrated to 400 ng µl -1.

### Creation of a *B. breve* UCC2003 Transposon Mutant Bank

*B. breve* UCC2003 electrocompetent cells were prepared according to a previously described procedure [35], with the following minor modifications. The carbohydrates; glucose, lactose, Vivinal GOS or ribose, were adopted as the main energy and carbon sources, at 1% w/v final concentration, in the mMRS broth for the preparation of competent cells.

To prepare the transposome complex: 2 µl of the EZ::TN<TetW> transposon were incubated with 4 µl of purified Tn5 transposase (Epicentre Biotechnology, Madison, USA) and 4 µl of 50% (w/v) glycerol to give a total reaction volume of 10 µl in accordance with the manufacturer’s instructions, to produce transposition complexes, also called transposomes [36]. 0.2 µl (20 ng) of the assembled transposome was combined with freshly prepared electrocompetent cells of *B. breve* UCC2003 for a single electroporation experiment. Tet-resistant transformants were selected on RCA plates supplemented with tetracycline. Resulting colonies were manually inoculated into individual wells of 96-microwell plates containing 150 µl of RCM broth supplemented with tetracycline. Following overnight growth, wells were supplemented with glycerol (Sigma, St Louis, USA) to a 20% w/v final concentration, and plates were stored at −80°C.

To prepare the mutant library of *B. breve* UCC2003, 32 independent electroporation experiments were performed, preparing competent cells following growth in mMRS broth supplemented with 0.05% L-cysteine and 1% w/v lactose as the sole carbon source. Following electroporation cells were recovered in RCM, containing both glucose and starch as carbon sources, and incubated anaerobically at 37°C for 30 minutes, which is not long enough for individual transformants to commence growth on the recovery medium thus minimizing the chance of isolating clonal siblings.

### Southern Hybridisation Analysis and Determination of Transposon Insertion Sites

DNA from *B. breve* UCC2003-derived transposon insertion mutants was isolated according to previously described procedures [37]. Southern blot transfer of BstEII digested chromosomal DNA was performed according to Sambrook et al. [32]. A PCR product encompassing *tetW* was used as the hybridisation probe and the hybridization and detection were performed according to the protocol of the digoxigenin DNA-labelling and detection kit (Roche Molecular Biochemicals, Lewes, United Kingdom).

Inverse PCR (i-PCR) reactions were performed to determine the transposon insertion site in selected tetracycline-resistant *B. breve* transformants. Two µg of chromosomal DNA samples were restricted with BstEII, cleaned by phenol extraction (1∶1) and ethanol precipitated. Amounts of 0.2 and 0.4 µg of the restricted DNA were self-ligated in a final volume of 50 µl using T4 DNA Ligase (Promega, Wisconsin, USA). Ligation reactions were incubated overnight at room temperature. Following ligation, DNA samples were phenol extracted, ethanol precipitated and resuspended in 10 µl of distilled water. Half of this sample was then used as template for the inverse-PCR reactions that were performed with the primers i-PCR-Fw and i-PCR-Rev (Table 1). PCR products were sequenced with the nested primers pMOD-fw-seq and pMOD-rev-seq (Table 1) to allow determination of the precise location of a transposon insertion.

### Phenotypic Screening and Monitoring of Carbohydrate-dependent Growth

*B. breve* UCC2003-Tn5 insertion mutants from 96-well plates were manually replicated using a 96-pin replicator (Boekel Scientific, Pennsylvania, USA). Stocked clones from the −80°C freezer were transferred to fresh microwell plates containing RCM supplemented with tetracycline (10 µg ml-1) and were incubated overnight, anaerobically at 37°C. Bacterial mutants were subcultured twice in RCM with tetracycline and subsequently fresh overnights were spotted onto QTrays (Molecular Devices, Berkshire, United Kingdom) containing either RCA or mMRS Agar, supplemented with 0.05% w/v final concentration of L-cysteine and 0.5% w/v final concentration of D-glucose, D-lactose, D-galactose or pullulan. The QTrays were incubated anaerobically at 37°C for 48 hours. Transposon mutants which grew on RCA but failed to grow in mMRS containing a single carbohydrate as the sole carbon and energy source were selected for further analysis.

Carbohydrate-dependent growth of selected mutants was confirmed through inoculation into liquid mMRS broth containing 0.5% w/v of the following carbohydrates: D-glucose, D-lactose, D-galactose or pullulan. Where relevant for comparison, D-glucose or D-ribose was employed as a positive control for mutant growth on mMRS-based broth. OD600nm was monitored during 24 hours of incubation at 37°C under anaerobic conditions. Subsequent identification of the transposon insertion site in selected mutants was performed as stated above.

## Results

### Optimization of Transposition Conditions in *B. breve* UCC2003

Since the successful application of the EZ::TN transposome strategy to generate significant numbers of insertion mutants is critically dependent on the transformation efficiency of the competent cells, both transformation efficiencies (following electroporation of a replicative vector, pAM5, isolated from *B. breve* UCC2003), and transposition frequencies (following electroporation of 20 ng of EZ::TN transposome) were determined on various batches of competent cells. Initially, the EZ::TN transposome strategy was shown to have limited success when the transposition complexes were introduced by electroporation into *B. breve* UCC2003 competent cells freshly prepared according to the standard procedure based on growth in mMRS broth containing glucose as the sole carbon source [35]. Under these experimental conditions the transformation efficiency for pAM5 plasmid DNA was 8×10^6^ CFU µg^−1^ DNA, which is within the range of previously described values for the same strain [21]. However, the number of transposon mutants was too low for a practical application of the EZ::TN system (Table 2); therefore an optimization of the method to increase the transformation efficiency and/or the transposition frequency was necessary.

**Table 2:** Transformation efficiencies and transposition frequencies.

Sugar	Transformation Efficiency*	Transposition Events*
Glucose	8.24×10^6^	1±0.00
GOS	1.47×10^7^	38±14.14
Ribose	1.2×10^7^	198±19.80
Lactose	1.38×10^7^	1554±192.33
Ribose+Lactose	2.19×10^7^	986±123.04

* Transformation efficiencies and transposition frequencies were determined following electroporation of a replicative vector, namely pAM5, or a tetracycline-resistant TN5 transposome, respectively, into *B. breve* UCC2003 competent cells grown in different carbohydrates. Transposition events are average of independent duplicates.

The inclusion of different carbon sources in the growth medium that was used to prepare the competent cells only slightly modified the transformation efficiencies obtained, but significantly improved the transposition frequencies (Table 2). In particular, when electrocompetent cells were prepared from cells that had been grown in mMRS containing lactose as the sole carbohydrate source higher amounts of transposon insertion mutants were obtained per electroporation reaction (up to 1,500 putative transposon insertion mutants per 20 ng of transposome electroporated in a single electroporation attempt). This transposition frequency is high enough to routinely perform random mutagenesis in bifidobacteria and is significantly higher than the reported frequencies achieved for this Tn5-based methodology in other bacteria considered recalcitrant to transformation [38]. Therefore, lactose was selected as the carbon source to be used to prepare competent cells to create a comprehensive bank of *B. breve* UCC2003 transposon mutants. Utilization of RCM, which contains both glucose and starch as carbon sources, plus tetracycline as the transposon mutant selection medium ensured that mutants defective in lactose metabolism could still be represented in the bank. The applicability of this transposon mutagenesis strategy for other bifidobacteria was also tested in a second strain, namely *B. breve* NCFB2258, in which a maximum amount of 700 tetracycline resistant clones was obtained per electroporation reaction when applying the conditions optimized for *B. breve* UCC2003. However, it stills remains to be determined whether further optimization of the procedure might lead to increase transposition frequencies in the strain NCFB2258.

### Confirmation of Transposition Insertion Events

**Figure 1:**
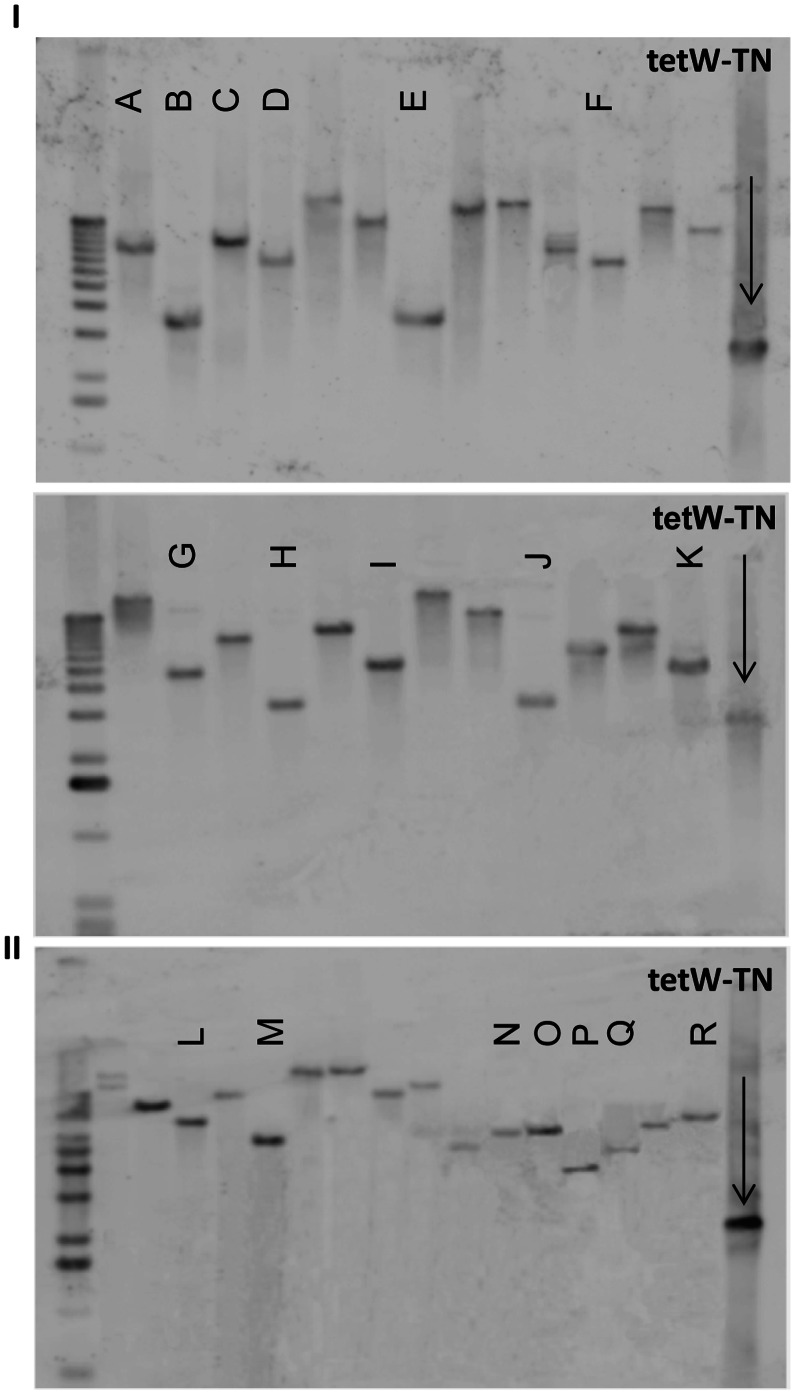
Confirmation of transposon insertion events into *B.* *breve* UCC2003 and *B. breve* NCFB2258 genome by Southern hybridization. Blots of twenty-six randomly selected mutants of *B. breve* UCC2003 (Panel I, two blots) and sixteen randomly selected mutants of *B. breve* NCFB2258 (Panel II, single blot) are shown. Lanes labeled from A to R correspond to mutants whose insertion site was subsequently sequenced as indicated in Table 3.

For confirmation of the expected transposition events in the *B. breve* strains, Southern hybridization analyses were performed. Chromosomal DNA preparations, extracted from randomly selected transposon-generated, tetracycline-resistant mutants (70 mutants in the case for *B. breve* UCC2003 and 16 mutants for *B. breve* NCFB2258), were BstEII digested, after which Southern analysis was performed using the tetracycline-resistance gene (*tetW*) as a probe. The blots revealed the presence of the transposon, in single copy, on the chromosome of the selected tetracycline-resistant transformants (examples are shown in Fig. 1). The size of hybridizing bands appeared to be different among the majority of the mutants, suggesting that transposon insertion had occurred at different positions within the chromosome. Sequencing of inverse-PCR products and comparison to *B. breve* UCC2003 genome information enabled the identification of the transposon insertion point for a selection of clones. Our sequencing efforts focused on those hybridizing bands showing apparently similar sizes on the blots to demonstrate that these represented individual transposon insertions in different chromosomal locations (corresponding to lanes A through to R in Fig. 1). The transposon site mapping experiments confirmed that insertions had occurred at different positions (Table 3), consistent with random transposon-mediated mutagenesis. In addition, the presence of insertions located at different positions within the same gene (Table 3) increase the probability of finding disruptive mutations for a given gene. Furthermore, the presence of 9 bp duplications in the chromosomal regions flanking the inserted transposon at the identified target sites, generated following repair of the chromosomal gaps created during the transposition event, is further evidence of Tn5-mediated transposition [33], [39].

**Table 3:** Mapping transposon insertion sites in a random selection of transposon insertion mutants.

Clone ID	Point of insertion	9bp duplication	Gene	Predicted Function
***B. breve*** ** UCC2003**				
**A**	409756	ctgcagggc	Bbr_0296	Alpha-amylase family protein
**B**	2084087	ccgcatgcc	Bbr_1690	ebgA-Evolved beta-galactosidase α-subunit
**C**	1884781	gcctgggcg	Bbr_1512	Transposase
**D**	1802786	catcgcagg	Bbr_1445	Activator of (R )-2-hydroxylglutarylCoA dehydratase
**E**	492623	cagttaccg	Bbr_0366	Conserv. Hypothetical secreted protein
**F**	1527997	gtacacgac	Bbr_1222	Phosphohydrolase (MuT/nudx fam prot)
**G**	1719332	ccccggagg	Bbr_1383	Conserv. Hypothetical protein with DUF domain
**H**	502520	gccgacgtc	Bbr_0375	Conserv. Hypothetical membrane spanning protein
**I**	633832	cccgatgac	Bbr_0482	Conserv. Hypothetical protein
**J**	1771618	gatgaggtt	-	223 nt upstream Bbr_1420
**K**	634533	gttcacgcg	Bbr_0482	Conserv. Hypothetical protein
**UCC1**	1167747	gataagaag	Bbr_0915	Conserv. Hypothetical protein with DUF558 domain
**UCC3**	325241	ggtctgtgt	Bbr_0230	Phage infection protein pip2
**UCC6**	15851	agaccactg	-	intergenic region Bbr_0010-Bbr_0011
**UCCIE1**	1643659	cctctaccg	Bbr_1316	Transporter, MFS Superfamily
**UCCIE12**	590969	gttttacgc	Bbr_0444	Membrane spanning polysaccharide biosynthesis protein
**UCCIF6**	2090095	accttgaaa	Bbr_1693	Glycosyl hydrolases family 65, Kojibiose phosphorylase
**UCCIF12**	1022428	ccggaagcc	Bbr_0787	pfl Formate acetyltransferase
**UCCIH12**	850699	cgttggtag	Bbr_0651	Conserved hypothetical secreted protein
**UCCIIC1**	1795092	gaacaagcc	Bbr_1442	bgl2 Beta-glucosidase
**UCCIIE8**	1349191	gtacgacgt	Bbr_1084	Conserved hypothetical protein with helix-turn-helix motif
***B. breve*** ** UCC2003 selected following carbohydrate-utilization screening**				
**101C6**	14835	gctgaggcc	Bbr_0010	lacZ1 Beta-galactosidase
**163D6**	647753	gtgtcccgc	Bbr_0491	galT1Galactose-1-phosphate uridylyltransferase
**181D10**	648238	gccgtacat	Bbr_0491	galT1 Galactose-1-phosphate uridylyltransferase
**101C4**	181945	acttcctga	Bbr_0121	Conserved hypothetical membrane spanning protein
**164B7**	182029	tggccttcc	Bbr_0121	Conserved hypothetical membrane spanning protein
***B. breve*** ** NCFB2258 clones**				
**L**	-	cagggccac	-	Transcriptional regulator LacI family
**M**	-	tcggtgaac	-	Homolog to Maf protein
**N**	-	gggatgtgg	-	Beta-glucosidase
**O**	-	gtgtagaga	-	Permease protein of ABC transporter for sugars
**P**	-	gcccagaac	-	Permease protein of ABC transporter for metals
**Q**	-	ggccgccac	-	Conserved hypothetical protein
**R**	-	aggccaccg	-	Homolog to ABC transporter, ATP binding component

### Creation of a *B. breve* UCC2003 Transposon Mutant Library

Comprehensive libraries of random mutants provide a convenient collection of mutants in almost any non-essential gene, allowing functional screens on a genome-wide scale to be performed. Ideally, mutants should contain a single insertion within a given genome, the distribution of the insertions should be random across the genome and the size of the mutant’s collection should be large enough to guarantee that every non-essential gene would be targeted in at least one clone in the collection [40]. Therefore, in view of the transposition frequencies achieved in strain *B. breve* UCC2003, the conditions optimized in the above examinations were employed to create a bank of random mutants in *B. breve* UCC2003, which comprised of approximately 20,000 transposon insertion mutants. In a simplified model, assuming that the transposon is randomly inserted and that all the genes have the same probability of being the target of a mutation, the probability of finding a mutation within a particular gene might be defined as P = 1-(1-X/G)^n^, where P is the probability of finding a mutation within a particular gene, X is the average length of the genes, G is the genome length and n is the number of clones within the library [41]. Therefore, based on *B. breve* UCC2003 genome information [18], the probability of finding a mutation within a given gene in our mutant library is 99.99%. This formula does not take into consideration the presence of essential genes on a bacterial genome for which a viable mutant would never be recovered, or indeed the occurrence of genes as part of operons, in which any insertion would inactivate all genes located downstream in the same operon. Thus, the realistic probability of finding at least one mutant carrying a mutation in a non-essential gene of *B. breve* UCC2003 in our mutant bank is likely to be very close to 100%.

### Screening for Defective Growth in Selected Carbon Sources, Identification of Transposon Insertion Sites and Phenotypic Confirmation

**Figure 2:**
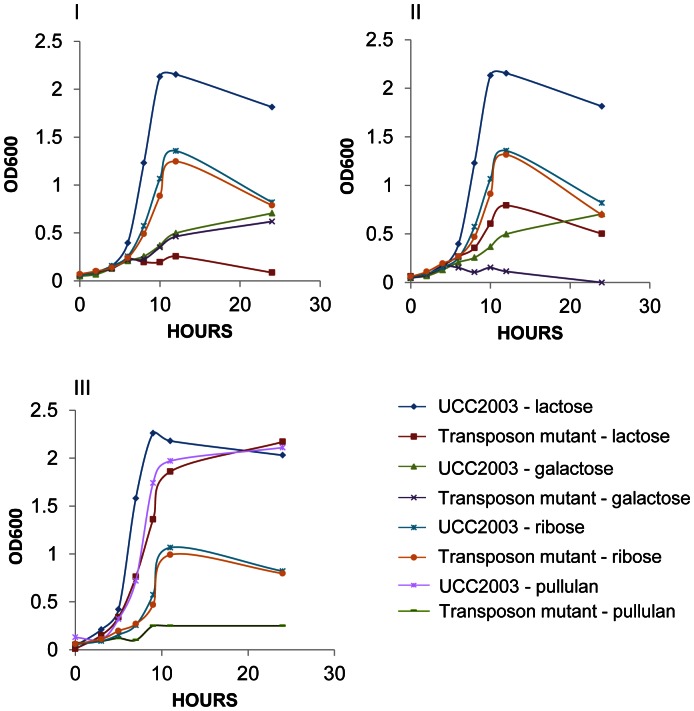
Growth profiles of *B.* *breve* UCC2003 and derived transposon mutant strains, 101C6 (I), 181D10 (II) on lactose, galactose and ribose; and 164B7 (III) on lactose, pullulan and ribose. Presented data are average of duplicate independent growth experiments.

**Figure 3:**
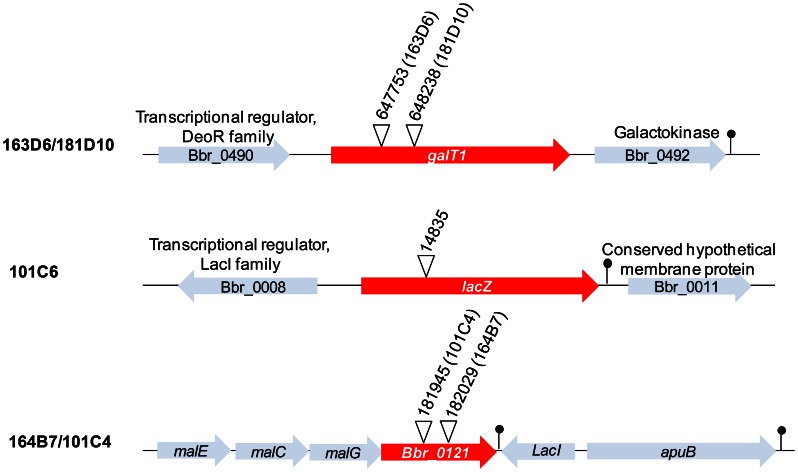
Genomic position and surrounding regions of the insertion site of a number of transposon insertion mutants that were isolated based on their inability to grow on one or more carbohydrates. The diagram was drawn to scale using *B. breve* UCC2003 genome sequence information. White open arrowheads represent the location of the transposon with the specific location indicated as the genome coordinate based on accession number CP000303. Red arrows represent transposon-disrupted open reading frames. Grey arrows represent flanking open reading frames. Lollipops indicate transcriptional terminators predicted by ARNold Web server (http://rna.igmors.u-psud.fr/toolbox/arnold/).

Comprehensiveness of the *B. breve* UCC2003 transposon mutant library was tested by conducting a phenotypic screen to identify clones defective for growth on particular carbon sources (D-lactose, D-galactose and pullulan). A total number of 3,744 mutants from the mutant library were screened for an inability to grow on mMRS when supplemented with a particular carbon source (agar plates containing 0.5% w/v of lactose, galactose, or pullulan). This phenotypic screening was followed by validation of the observed growth deficiency of selected clones into liquid mMRS broth supplemented with the corresponding carbon source. The transposon insertion site was determined for five mutants, which exhibited a particular phenotype of failing to grow in mMRS supplemented with one or more of the tested carbohydrates in both agar and broth assays (Fig. 2, Table 3). From those, one mutant, designated 101C6, showed absence of growth as determined by final OD600 after 24 hours of incubation on lactose (Fig. 2 I). Mutant 101C6 was shown to harbor the transposon within a gene (Bbr_0010) encoding one of the previously identified β-galactosidases in *B. breve* UCC2003 (Table 2, Fig. 3) [42]. Our data therefore indicates that the β-galactosidase encoded by Bbr_0010 is the main β-galactosidase employed by this bacterium to metabolize lactose, at least under the conditions tested.

Two mutants, namely 181D10 (Fig. 2 II) and 163D6, which showed impaired growth on lactose and absence of growth in galactose, were found to harbor the transposon inserted within a predicted homolog of the *galT* gene (Bbr_0491) (Table 2, Fig. 3). This gene is predicted to encode the enzyme that catalyzes the second step of the Leloir pathway, converting D-galactose into UDP-glucose, which would subsequently be fed into the bifid shunt [43], [44]. Accordingly, these mutants also showed reduced growth abilities on D-lactose which is expected to be due to their inability to metabolize the galactose component of the disaccharide lactose.

Finally, two mutants, 164B7 (Fig. 2 III) and 101C4, were identified to be deficient in growth on the polysaccharide pullulan. In both mutants the transposon insertion was mapped to different positions within the same gene (Bbr_0121), encoding a component of a predicted maltodextrin ABC-type transporter, and located upstream of the previously described amylopullulanase-encoding gene Bbr_0123 (Table 2, Fig. 3) [45]. The product encoded by this gene is predicted to be involved in the uptake of the maltodextrins cleaved as a result of amylopullulanase activity on pullulan in the extracellular environment. Finally, it is worth noting that all mutants discussed above were shown to grow equally well as their wild type UCC2003 derivative on mMRS supplemented with either ribose (Fig. 2) or glucose (results not shown).

## Discussion

The expanding collection of complete *Bifidobacterium* genomes coupled to extensive efforts to expand the molecular toolbox for this group of bacteria, are contributing to make bifidobacteria more accessible to genetic manipulation [46], [47]. However, only a few targeted mutations have been reported in certain strains [22], [45], [48], [49] and no systems for random mutagenesis in bifidobacteria have yet been described. In this work a *B. breve* UCC2003 TN5 insertion library consisting of nearly 20,000 mutants was created based on the EZ::TN transposome system. Further analysis of randomly selected clones as well as analysis of clones selected following a screening for the inability to grow on certain carbohydrates, has validated the library since the transposon insertions were proved to be single and apparently random. This transposon mutagenesis strategy was also successful in a second strain of *B. breve* and may thus be applicable to other bifidobacterial strains and species, provided that high transformation efficiencies and antibiotic selection markers are available.

Transposon mutagenesis systems have been extensively employed in Gram negative bacteria and more recently, also in Gram positives, including certain Actinobacteria [50], [51], [52]. However, implementation of *in vivo* transposon delivery systems generally requires conditional origins of replication and efficient determinants to control the transposase expression, which are not readily available for most *Bifidobacterium* strains [47]. The EZ::TN Transposome system, based on the *in vitro* assembly of stable complexes between a purified transposase and a transposon which are directly introduced into the host by electroporation, bypasses some of these limitations [36]. Furthermore, this system has been successfully implemented for a wide range of bacteria, including a significant number of Actinobacteria [53], [54], [55], [56], [57]. Low transformation efficiencies, endogenous restriction endonucleases and the efficient expression of the selection marker within the transposon, are the main bottlenecks for the effectiveness of the EZ::TN Transposome system [58]. In this work, the use of a selection marker of bifidobacterial origin, *tetW* [31], which doesn’t harbor the restriction sites that *B. breve* UCC2003 cleaves [21], allowed us to create the first transposon insertion mutants in bifidobacteria. In addition, optimization of competent cell preparation resulted in transposition frequencies high enough to routinely perform transposon-based random mutagenesis in *B. breve* UCC2003 (Table 2). The transposition frequency values obtained in this work for bifidobacteria are within the same range as those obtained for other bacteria including *Rhodococcus* [50] or *Mycobacterium* [55], although transformation efficiencies reported for bifidobacteria are still lower than those reported for other bacteria. Remarkably, transformation efficiencies reported in *Lactobacillus casei* were 10-fold higher than values achieved in *B. breve* UCC2003 although transposition frequencies were around 10-fold lower [38]. This suggests that further optimization of competent cells preparation in other bacteria may allow dramatic increases in transposition efficiencies as demonstrated in this work for bifidobacteria. However, the effect of host-encoded restriction endonucleases and differential efficiencies on expression of selective markers cannot be ruled out when performing transposon mutagenesis with this transposome strategy into new strains. Further knowledge on specific host-encoded restriction endonucleases will undoubtedly facilitate transposon design for additional bifidobacterial species/strains.

In this work the generated mutant library for *B. breve* UCC2003 represents an estimated average coverage of ∼10 insertions per gene. The size of this bank is within the range of other bacterial mutant collections and is thus presumed to contain insertions in the vast majority of non-essential genes of *B. breve* UCC2003. Therefore, a phenotypic screening was conducted to confirm the usefulness of this *Bifidobacterium* collection of mutants to perform functional genomic analysis. Utilization of different carbohydrates was selected as a proof-of-principle screening due to the large amount of information that extensive research on this topic has revealed in *B. breve* UCC2003 over the past few years [44]. Although only ∼20% of the mutants present in the bank were screened, clones showing inability to grow were found for the three tested carbohydrates. In addition, a number of mutants were identified that harbour insertions at different positions within the same gene, thus confirming that multiple, independent insertions per gene are present in the mutant bank. Therefore, this is expected to increase the probability of finding insertions within a given gene, at positions sufficiently disruptive to cause phenotypic alterations. Furthermore, the identified transposon insertion sites of analyzed mutants could be correlated with the observed phenotypes, demonstrating the usefulness of this mutant library for functional screenings. However, one has to keep in mind that polar effects of the inserted transposon on downstream-located genes within a single transcriptional unit cannot be excluded and complementation experiments will be necessary to ascertain whether an interrupted gene is directly responsible for an observed phenotype.

In conclusion, the first library of random mutants of a *Bifidobacterium* strain was obtained. Preliminary analysis of transposon insertion mutants and a proof-of-principle screening, led us to validate the usefulness of the bank to perform efficient genome-wide screenings. The transposon mutagenesis system was also successfully applied to a second *B. breve* strain; however, further research is needed in order to establish if this mutagenesis tool can be extended to other bifidobacterial strains that currently seem to be recalcitrant to genetic manipulation. Construction and optimization of a second generation TN5-TetW-based transposon system aimed at tracking mutants within pools or *in vivo* models will further facilitate functional genomic efforts in bifidobacteria including the elucidation of molecular mechanisms of probiotic action.
